# Mycotic Aneurysm of the Infrarenal Abdominal Aorta Caused by Campylobacter fetus Identified After 10-Day Antibiotic Therapy

**DOI:** 10.7759/cureus.85579

**Published:** 2025-06-08

**Authors:** Takehiro Hashimoto, Yoshihide Hioki, Kosaku Komiya, Kazufumi Hiramatsu

**Affiliations:** 1 Infection Control, Oita University Hospital, Yufu, JPN; 2 Respiratory Medicine and Infectious Diseases, Oita University Faculty of Medicine, Yufu, JPN

**Keywords:** 16s rrna gene sequencing, campylobacter fetus, culture–negative specimens, infrarenal abdominal aorta, mycotic aortic aneurysm

## Abstract

We report a rare case of a mycotic aneurysm caused by *Campylobacter fetus*, undetectable by Gram staining or surgical specimen culture obtained after 10 days of antimicrobial therapy. However, the pathogen was successfully detected by 16S rRNA gene sequencing. An 83-year-old woman with comorbid hypertension presented with lumbar and lateral abdominal pain and was diagnosed with an abdominal mycotic aneurysm. Despite its low sensitivity for a negative Gram staining test and culture-negative specimens, 16S rRNA gene sequencing can identify the causative organism and facilitate appropriate antibiotic therapy.

## Introduction

Mycotic aneurysms are rare, accounting for approximately 0.7% to 1.3% of all aortic aneurysms [1]. In certain cases, they progress more rapidly than other noninfectious aortic aneurysms, potentially expanding within days [2]. *Campylobacter fetus *exhibits a tropism for vascular tissue, particularly the abdominal aorta, where it can cause rapidly expanding aneurysms with a high risk of rupture [3]. The standard treatment involves targeted antibiotic therapy alongside surgical resection, debridement of the infected tissues, and graft replacement [4]. Prolonged postoperative antibiotic therapy, typically six to 12 months, is often recommended, irrespective of the surgical method used [5]. However, long-term use of antibiotics poses a risk of the emergence of drug-resistant bacteria, underscoring the need to select an appropriate regimen based on blood and infected tissue culture results and antimicrobial susceptibility testing of microorganisms. In some cases, antibiotics are initiated prior to obtaining cultures and performing surgical intervention, rendering the causative microorganisms unidentified. We report a case of an infected abdominal aortic aneurysm caused by *C. fetus* that was undetectable by Gram staining of the surgical specimen obtained after 10 days of antimicrobial therapy. However, it was successfully identified using 16S rRNA gene sequencing, facilitating the selection of an appropriate antibiotic regimen.

## Case presentation

An 83-year-old woman with comorbid hypertension presented to her former physician with chief complaints of lumbar and lateral abdominal pain. She is a never-smoker. A plain abdominal CT revealed an abdominal aortic aneurysm, and imminent rupture was suspected; therefore, she was referred to our hospital. At the time of admission, her body temperature was 37.0°C, indicating the absence of high fever. Laboratory test results on admission revealed a prominent elevation in WBC count (20,880/μL) and C-reactive protein (CRP) level (15.20 mg/dL) (Table 1). Echocardiography revealed no vegetation.

**Table 1:** Laboratory data on admission

Parameter	Result	Normal range
White blood cells (× 10^3^/μL)	20.88	3.3–8.1
Neutrophils (%)	88.9	40.5–76.5
Lymphocytes (%)	6.8	15.2–48.4
Monocytes (%)	4	2.0–8.9
Eosinophils (%)	0.1	0.0–7.7
Basophils (%)	0.2	0.0–0.9
Red blood cells (× 10^6^/μL)	3.46	3.86–4.92
Hemoglobin (g/dL)	10.7	11.6–14.8
Hematocrit (%)	32.2	35.1–44.4
Platelets (× 10^4^/μL)	20.2	15.8–34.8
Total protein (g/dL)	7.18	6.6–8.1
Albumin (g/dL)	2.94	4.1–5.1
Total bilirubin (mg/dL)	0.56	0.4–1.5
Aspartate aminotransferase (IU/L)	44.1	13–30
Alanine aminotransferase (IU/L)	47.8	7–23
Alkaline phosphatase (U/L)	74	38–113
γ-Glutamyl transpeptidase (IU/L)	28	9–32
Lactate dehydrogenase (mg/dL)	198	124–222
Creatine kinase (U/L)	98	41–153
Blood urea nitrogen (mg/dL)	22.5	8–20
Creatinine (mg/dL)	1.53	0.46–0.79
Serum sodium (mEq/L)	137	138–145
Serum potassium (mEq/L)	3.8	3.6–4.8
Serum chloride (mEq/L)	107	101–108
Serum calcium (mg/dL)	8.6	8.8–10.1
Serum phosphate (mg/dL)	2.8	2.7–4.6
C-reactive protein (mg/dL)	15.2	0.0–0.14

**Figure 1:**
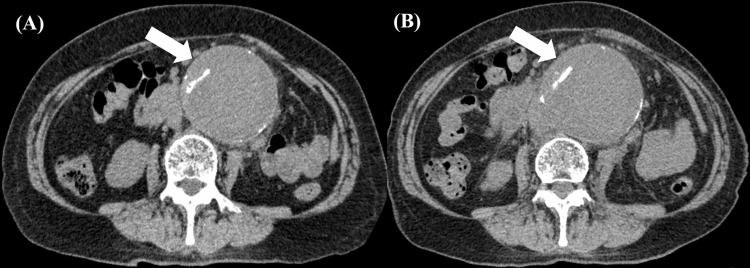
Axial abdominal CT scans A: On admission, a mycotic aneurysm of the infrarenal abdominal aorta (white arrow) was observed (size: 73 mm × 78 mm); B: On day five, the mycotic aneurysm was found to be enlarged (white arrow) despite antibiotic therapy (size: 81 mm × 85 mm)

**Figure 2:**
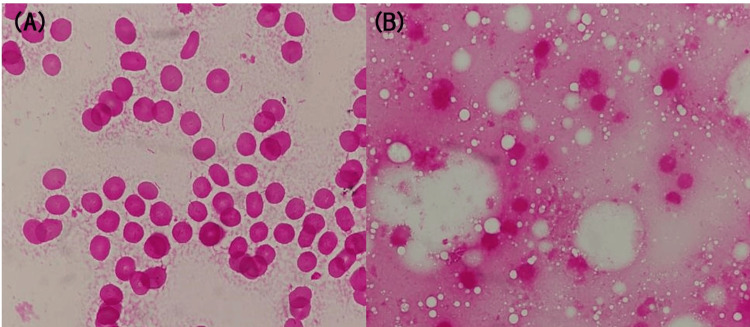
Gram staining of blood cultures and aortic wall tissue A: Gram staining of blood cultures showed gram-negative curved rods (1000× magnification); B: Gram staining performed on the aortic wall tissue revealed no bacterial presence (1000× magnification)

The contrast-enhanced CT scan at our hospital showed no evidence of rupture (aneurysm size: 73 mm × 78 mm) (Figure 1A) and in the absence of emergency surgical intervention, antimicrobial therapy with meropenem (MEPM) at 1g/day was initiated, considering renal function after obtaining two sets of blood cultures. By day three, gram-negative curved rods were isolated from both blood cultures (Figure 2A), which were later identified as *C. fetus *subsp.* fetus *using matrix-assisted laser desorption/ionization time-of-flight mass spectrometry (Bruker Daltonics, GmbH, Bremen, DEU); score value: 2.392. On day five, because the patient reported abdominal pain, a plain CT scan of the abdomen was performed, which revealed increased fatty tissue density around the aortic aneurysm and enlargement of the aortic aneurysm (81 mm × 85 mm) (Figure 1B), raising suspicion of an infected aortic aneurysm. The minimum inhibitory concentration values of the organism determined by using the broth microdilution method were as follows: ampicillin 2 µg/mL; cefotiam 8 µg/mL; cefotaxime 8 µg/mL; meropenem ≤ 0.12 µg/mL; clarithromycin 1 µg/mL; clindamycin 1 µg/mL; levofloxacin 1 µg/mL; and minocycline 1 µg/mL. On day seven, the treatment regimen was revised to ampicillin (ABPC). On day 10, laboratory test results revealed minimal improvement in inflammatory markers, i.e., the WBC count was 15,250 /μL and the CRP level was 18.1 mg/dL; the patient complained of severe abdominal pain. Therefore, the patient underwent Y-graft replacement surgery and omental packing to manage the infection and identify causative organisms on day 10 of hospitalization. Gram staining of the postoperative tissue specimens revealed the presence of polymorphonuclear leukocytes, but no bacteria (Figure 2B), and tissue specimen cultures were negative.

The collected samples were incubated under various conditions: on 5% sheep blood agar (Nissui Pharmaceutical Co., Tokyo, JPN) and chocolate agar (Kyokuto Pharmaceutical Industrial Co. Ltd., Tokyo, JPN) in aerobic environments; on Brucella HK agar (Kyokuto Pharmaceutical Industrial Co. Ltd., Tokyo, JPN) under anaerobic conditions; and on HK semisolid medium (Kyokuto Pharmaceutical Industrial Co. Ltd., Tokyo, JPN) in aerobic settings. Despite these diverse cultivation efforts, no colony formation was detected on any of the solid agar media. To identify the causative organisms of the mycotic aneurysms, 16S rRNA gene sequencing was performed on the specimen. Total DNA was extracted using the Cica Geneus DNA Extraction Kit (Kanto Chemical Co. Inc., Tokyo, JPN), according to standard protocols. The 16S ribosomal RNA gene sequencing was conducted using the primers 518A (5'-CCAGCAGCCGCGGTAATAC-3'), 786B (5'-GACTACCAGGGTATCTAATC-3'), and 907A (5'-AAACTCAAAGGAATTGACGG-3'). Nucleotide sequencing was carried out using the BigDye Terminator v3.1 Cycle Sequencing Kit (Thermo Fisher Scientific, Waltham, MA, USA), and the resulting data were analyzed using a SeqStudio Genetic Analyzer (Thermo Fisher Scientific). A basic local alignment search tool (BLAST) search of the obtained sequence identified the bacterium as *C. fetus*, with 95% sequence similarity (1407 of 1481 nucleotides) to *C. fetus* subsp. *fetus *(GenBank accession number CP072664.1). Blood cultures were negative on day eight and day 12. Postoperative antibiotic therapy with ABPC was continued, and on day 43, the patient was switched to amoxicillin, which was continued for six months. On day 44, the patient was transferred to another hospital for rehabilitation. The patient has been stable without recurrence of the mycotic aneurysm for two years after discharge.

## Discussion

Mycotic aneurysm is a rare but serious condition characterized by an infected arterial wall, leading to localized dilation and an increased risk of rupture. The infection weakens the arterial wall and may result from underlying conditions such as endocarditis, septicemia, or intravenous drug abuse [1,2,5]. The most common symptom is pain (77%), followed by fever (67%) [5]. The most common pathogens isolated in mycotic aneurysms are *Salmonella* spp. (33.4%), *Staphylococci* spp. (15.6%),* Streptococci* spp. (10.4%), and *Escherichia coli* (3.1%) [5]. In contrast, mycotic aneurysms caused by *Campylobacter* species are rare [5].

*Campylobacter* spp. are gram-negative, mobile, curved rods, most commonly transmitted through the consumption of undercooked meat and unpasteurized milk and less commonly through direct contact with domestic animals [6]. The major *Campylobacter* species associated with human diseases include *Campylobacter jejuni*, *Campylobacter coli*, *Campylobacter upsaloensis*, *Campylobacter lari*, and *C. fetus* [7]. *Campylobacter jejuni*, *C. coli*, and *C. upsaloensis *primarily cause gastrointestinal infections and are linked to secondary conditions such as Guillain-Barre syndrome and reactive arthritis [7-9]. *Campylobacter lari* is associated with bacteremia and gastrointestinal and urinary tract infections [7,8], while *C. fetus *commonly cause primary infections such as bacteremia and extraintestinal infections, and may also cause secondary infections such as mycotic aneurysms and thrombophlebitis [7,8]. *Campylobacter fetus* accounts for 1% of *Campylobacter* spp. infections [10]. Published data on antimicrobial susceptibility patterns of this organism are limited, and no established interpretive criteria are currently available [11]. Reports have suggested the efficacy of ampicillin in the treatment of *C. fetus* infections. However, ampicillin susceptibility testing in *C. fetus *yielded minimum inhibitory concentration (MIC) values between 0.03 and 16 µg/mL, with inconsistent MIC90 results [12-14].

In the present case, despite appropriate antibiotic therapy, the patient exhibited continued aneurysm enlargement and limited clinical improvement. Consequently, surgical intervention was performed on day 10 of hospitalization. While mycotic aneurysms are typically attributed to a single pathogen, polymicrobial infections, though rare, have been documented [15]. Co-infections involving *Campylobacter* sp. and other enteric pathogens have been previously reported [16-18]. Consequently, specimens for cultures were obtained from the infected arterial wall for microbiological analysis. Gram staining of the aortic wall tissue showed no bacteria, and although culture results remained negative, 16S rRNA gene sequencing successfully identified *C. fetus*. Although 16S rRNA sequencing showed a higher positive rate in specimens with a positive Gram staining result than in those with a negative Gram staining result, a 16.9% positivity rate was reported in specimens with a negative Gram staining result [19].

Perioperative mortality associated with mycotic aneurysms was historically high, ranging from 26% to 44% prior to 2000 [5]. Thus, in some cases, antibiotic therapy is initiated before collecting cultures and performing surgical intervention, which leads to the causative microorganisms being unidentified. Gene sequencing can be a valuable diagnostic tool when both cultures and Gram staining are negative. Given the lower sensitivity of 16S rRNA gene sequencing compared to species-specific polymerase chain reaction (PCR) methods, it is crucial to recognize that the organism identified may not be the sole pathogen or may not be the actual causative agent. The possibility of contamination, polymicrobial infection, or an infection at another site should be carefully evaluated, considering the culture results, imaging studies, and clinical features [20,21].

The gold standard treatment for mycotic aneurysms involves a combination of antibiotics with resection, debridement of the infected tissues, and graft replacement [4]. Postoperative antimicrobial treatment is typically recommended for four to six weeks. However, owing to the significant risk of recurrent infection, along with the high rates of associated complications and mortality, and considering that many patients may be unfit for further major surgical interventions, extending antibiotic therapy for six to 12 months postoperatively is advantageous, regardless of the surgical approach followed [5,22].

Data on the efficacy of antibiotic therapy without surgical intervention remain limited. While antibiotics alone may help control infection, they are generally insufficient to prevent serious complications such as aneurysmal rupture [23]. However, in our case, the patient was treated with antibiotic therapy alone because of advanced age. Our experience, along with previous reports, suggests that conservative management may be considered in patients with high surgical risk [24]. However, if the aneurysm diameter expands or the clinical symptoms deteriorate, prompt surgical intervention should be considered.

## Conclusions

In our case, aortic wall tissue culture obtained during antibiotic therapy for the mycotic aneurysm of the infrarenal abdominal aorta was negative. However, we identified *C. fetus* as the causative organism via 16S rRNA sequencing, facilitating the selection of an appropriate antibiotic regimen for treating the infection. In cases where diseases require prolonged antibiotic therapy, identification of the causative organism through gene sequencing is valuable for determining the most appropriate antibiotic therapy.
